# Apatinib modulates sorafenib-resistant hepatocellular carcinoma through inhibiting the EGFR/JNK/ERK signaling pathway

**DOI:** 10.32604/or.2025.060407

**Published:** 2025-05-29

**Authors:** DEXUE FAN, WEI SU, ZHAOWEN BI, XINXING WANG, XIANWEN XU, MINGZE MA, LICHAO ZHU, ZHENHAI ZHANG, JUNLIN GAO

**Affiliations:** 1 Department of Hepatobiliary Surgery, Shandong Provincial Hospital Affiliated to Shandong First Medical University, Jinan, 250021, China; 2 Liver Gall Bladder and Pancreatic Surgery Ward, Qinghai Red Cross Hospital, Xining, 810001, China; 3 Department of Hepatobiliary Surgery, Shandong Provincial Hospital, Shandong University, Jinan, 250021, China; 4 Departments of Infectious Diseases, Shandong Provincial Hospital Affiliated to Shandong First Medical University, Jinan, 250021, China; 5 Department of Pediatric Surgery, Shandong Provincial Hospital, Shandong University, Jinan, 250021, China

**Keywords:** Apatinib, Sorafenib resistance, EGFR/JNK/ERK, Epithelial mesenchymal transformation, Hepatocellular carcinoma (HCC)

## Abstract

**Objectives:**

Apatinib has been reported to be a promising treatment for sorafenib-resistant hepatocellular carcinoma (HCC) patients. However, the underlying mechanism remains ambiguous. The study aimed to explore the efficacy of apatinib in sorafenib-resistant HCC and the underlying mechanism both *in vitro* and *in vivo*.

**Methods:**

After observing epithelial-mesenchymal transformation (EMT) changes in HepG2 and HepG2/Sorafenib cells, we treated them with varying concentrations of apatinib to assess its impact on sorafenib-resistant HCC. Subsequently, specific inhibitors of c-Jun N-terminal kinase (JNK, SP600125) and extracellular signal-regulated kinase (ERK, PD98059) were introduced to investigate whether apatinib influenced sorafenib-resistant HCC via modulation of the epidermal growth factor receptor (EGFR)/JNK/ERK signaling pathway *in vitro* and *in vivo*. Biological behavior changes were assessed through cell counting kit-8 (CCK-8), colony formation, transwell, and immunofluorescence tests. Simultaneously, Western blot analysis was conducted to elucidate the expression of proteins associated with EMT and the EGFR/JNK/ERK signaling pathway.

**Results:**

The HepG2/Sorafenib cells exhibited greater resistance to sorafenib compared to HepG2 cells, and sorafenib-resistant HCC was characterized by EMT changes. Apatinib demonstrated concentration-dependent inhibition of biological behaviors in HepG2/Sorafenib cells, with minimal impact on HepG2 cells. Additionally, apatinib had a pronounced effect on the expression of EMT-related proteins in sorafenib-resistant cells similar to that in sorafenib-sensitive cells. Furthermore, there was a dose-dependent reduction in the expression of proteins associated with the EGFR/JNK/ERK pathway in apatinib-treated groups. Notably, SP600125 and PD98059 contributed to the inhibition of EMT and EGFR/JNK/ERK pathway-related proteins by apatinib in sorafenib-resistant HCC.

**Conclusion:**

Apatinib potentially hindered the progression of sorafenib-resistant HCC by suppressing both EMT and the EGFR/JNK/ERK pathway.

## Introduction

Liver cancer, ranked as the fourth most prevalent malignancy, poses a significant threat due to its high mortality rates [1]. Notably, the liver lacks pain receptors (nerve endings), allowing changes within the organ, including the growth of small tumors, to occur without inducing direct pain sensations [2]. The liver’s substantial size and functional reserves enable it to maintain normal function despite partial occupancy by a tumor, leading to inconspicuous symptoms during the early stages [2]. Typically, hepatocellular carcinoma (HCC) remains asymptomatic and is often discovered incidentally during routine check-ups or liver disease monitoring, marking the subclinical phase [3]. However, the onset of HCC symptoms, such as liver pain, fatigue, weight loss, jaundice, and ascites, usually indicates the disease in its middle to late stages [4]. While surgery and radiotherapy are common clinical interventions, the cure rates for advanced stages remain low due to widespread cancer cell dissemination [5]. Recently, multiple studies have indicated that the Royal Marsden Hospital (RMH) score could serve as a readily available prognostic biomarker for cancer patients [6], however, its wide-scale implementation in clinical practice is still insufficient, and its true benefit in clinical decision-making has not been fully realized. Thus, the importance of early detection, diagnosis, and intervention for HCC cannot be overstated.

Sorafenib is a novel multi-targeted oral agent used in the treatment of inoperable or distantly metastatic HCC [7]. Since 2007, sorafenib has been approved to be the first-line therapy for advanced HCC; however, approximately just 30% of HCC patients benefit from treatment with sorafenib and these patients are inclined to develop resistance within 6 months [8]. The mechanisms underlying sorafenib resistance encompass alterations in cell signaling pathways, changes in drug targets, modifications in drug pumps and metabolism, variations in the tumor microenvironment, and evasion of drug feedback inhibition [8–10]. HCC stem cells inherently exhibit resistance to Sorafenib, impeding HCC neovascularization while intensifying local hypoxia and activating hypoxia signaling pathways [11,12]. This fosters epithelial-mesenchymal transition (EMT), and pseudoangiogenesis, ultimately promoting invasion and metastasis [13]. Additionally, multiple studies have reported the key roles of EGFR/JNK/ERK signaling in sorafenib resistance of HCC. For example, EGFR was highly expressed in sorafenib-resistant HCC cells [14]. An ERK inhibitor could enhance the sorafenib efficiency of HCC [15].

The development of resistance in HCC against sorafenib results in suboptimal clinical outcomes, necessitating the exploration of combination therapies involving other targeted drugs to enhance treatment efficacy. Apatinib, an inhibitor of VEGFR-2, has been reported to obviously improve overall survival in patients with advance HCC in a multicentre, double-blind, randomized, and placebo-controlled phase 3 trial [16]. Moreover, a phase II clinical trial showed that apatinib could be an encouraging first-line treatment in patients with advanced HCC [17]. Importantly, Zhang et al. have demonstrated that advanced HCC patients with sorafenib resistance achieved a longer overall survival after apatinib treatment [18]. These results indicated that apatinib could be a promising treatment for HCC patients with sorafenib resistance. However, the underlying mechanism has not been clarified. Therefore, in this study, the effectiveness of apatinib on sorafenib-resistant HCC and the relevant mechanism were investigated *in vitro* and *in vivo*.

## Materials and Methods

### Cell lines

HepG2 cells were procured from ATCC (Manassas, VA, USA) and maintained in an incubator with a culture medium consisting of 90% DMEM (PM150250, Procell, Wuhan, China), 10% FBS (10099141, GIBCO, Shanghai, China), and 1% penicillin/streptomycin (MA0110, Meilune, Dalian, China). No mycoplasma contamination was detected in the cell line.

### Establishment of sorafenib-resistant HepG2 cells

Sorafenib-resistant HepG2 cells were established according to a previous study [19]. The establishment process involved a gradual escalation of sorafenib exposure, starting at a concentration of 1 μM and increasing by increments of 1 μM every fortnight until the cells could tolerate the highest dose of 8 μM. Once the cells reached this tolerance level, they were maintained in a culture medium containing 8 μM sorafenib (HY-10201, MCE, Shanghai, China).

### Cell treatment

The HepG2 and HepG2/Sorafenib cells were treated with 0.001, 0.01, 0.1, 1 and 10 µM sorafenib for 24 h respectively to investigate the drug resistance. To assess the impacts of apatinib on sorafenib-sensitive and resistant HepG2 cells, the HepG2 and HepG2/Sorafenib cells underwent treatment with varying concentrations (0, 0.5, 1, 2, and 4 μM) of apatinib (S2221, Selleck, Shanghai, China) for 24 h. Subsequently, specific inhibitors of JNK (SP600125, Beyotime, Shanghai, China) and ERK (PD98059, Beyotime, China) were introduced to investigate the potential influence of apatinib on drug resistance in hepatoma cells through modulation of the EGFR/JNK/ERK signaling pathway. The cells were treated with 20 µM SP600125 or 10 µM PD98059 for 24 h.

### CCK-8 assay

The resistance of HCC to sorafenib and cell viability was assessed using the CCK-8 assay. Briefly, 5 × 10^4^ cells were seeded in a 96-well plate. The cells in different groups were treated with 0, 0.5, 1, 2, and 4 μmol/L of apatinib for 24 h, with the drug volume not exceeding one-tenth of the cell suspension volume. There were three replicate wells in each group. 10 μL of CCK-8 solution (G4103, Servicebio, Wuhan, China) to each well, and the cells were incubated for an additional 4 h. Finally, the optical density (OD) values at 450 nm were determined using a plate reader (multiskan sky, Thermo Fisher, Bothell, WA, USA).

### Immunofluorescence

The HepG2 and HepG2/Sorafenib cells were fixed with 4% paraformaldehyde (G1101, Servicebio, Wuhan, China) and 0.1% TritonX-100 (T8200, Solarbio, Beijing, China), followed by blocking with bovine serum albumin (G5001, Servicebio) for 15 min at room temperature. Subsequently, they were incubated overnight at 4°C with the anti-β-microtubulin antibody (1:400, 10068-1-AP, PTG, Rosemont, IL, USA). Afterward, visualization of microtubules in the HepG2 and HepG2/Sorafenib groups was achieved by incubating with the secondary antibody Multi-rAb CoraLite®® Plus 594-Goat Anti-Rabbit Recombinant Secondary Antibody (H + L) (1:500, RGAR004, PTG, USA) and observing under a fluorescence microscope (Eclipse C1, Nikon, Tokyo, Japan).

### Colony formation

After apatinib treatment, a colony formation assay was performed to investigate the effect of apatinib on the proliferation of HepG2 and HepG2/Sorafenib cells. Briefly, 1 × 10^3^ cells were seeded in a 6-well plate and cultured for about two weeks until visible clones were observed by the naked eye. Subsequently, these clones underwent fixation with 4% paraformaldehyde (1 mL/well, XW0130525894011, SinoPharm, Shanghai, China) for 60 min and stained with crystal-free crystal violet staining solution (500 µL/well, CB0331, Sangon Biotech, Shanghai, China) for 20 min. Finally, the clones were counted under an inverted microscope (CKX53, Olympus, Shanghai, China).

### Transwell assay

Invasion and migration assays of HepG2 and HepG2/Sorafenib cells were performed using a transwell assay kit (3422, Corning, Corning, NY, USA) according to previous studies [20]. Serum-free cell suspensions were prepared using DMEM culture solution without serum. 200 µL cell suspension was added into the upper chambers with a cell density of 1.2 × 10^5^/well. 500 µL DMEM supplemented with 30% FBS was added into the lower chamber. After 48 h, the migrated cells were fixed with 4% paraformaldehyde for 30 min, stained with 0.1% crystal violet (CB0331, Sangon, Shanghai, China) staining solution for 30 min, and observed under an inverted microscope (CKX53, Olympus) to evaluate their migration capability. Invasion experiments were conducted similarly to the migration experiments, with the exception that Matrigel (BD Biocoat 354234, Corning) spreading was applied at the bottom of the Transwell chambers.

### Western blot

The HepG2 and HepG2/Sorafenib cells were lyzed with the RIPA lysis buffer (AR0102, Boster, Wuhan, China) to extract the total proteins. Then the BCA Protein Assay Kit (P0012, Beyotime) was used to measure protein concentration. Proteins were separated and analyzed by SDS-PAGE at 80 V. After transferring to polyvinylidene fluoride (PVDF) membranes (IPVH00010, Millipore, Shanghai, China), proteins were treated with 5% skim milk for blocking and then incubated with primary antibodies against β-Tubulin III (Ab52623, Abcam, 1:5000), keratin (Ab8068, Abcam, 1:250), N-cadherin (Ab52623, Abcam, 1:1000), Vimentin (Ab52623, Abcam, 1:10000), p-EGFR (Ab32430, Abcam, 1:1000), p-JNK (9255, CST, 1:2000), p-ERK (4370, CST, 1:2000), EGFR (Ab52894, Abcam, 1:10000), JNK (9252, CST, 1:1000), ERK (4695, CST, 1:1000), and GAPDH (60004-1-Ig, Proteintech, 1:50000). Subsequent to incubation with HRP-labeled goat anti-mouse or rabbit secondary antibodies (ZB-2305/ZB-2301, 1:3000, ZSGB-Bio, China), protein bands were analyzed using Image Lab Software (Bio-Rad, Boulder, CO, USA). GAPDH was also used as an internal control.

### In vivo experiments

Sixteen four-week-old female Balb/c nude mice were obtained by Charles River (Beijing, China). After two weeks of adaptive feeding, HepG2/sorafenib cells were injected subcutaneously into the mice (1 × 10^6^ cells/mouse). Seven days later, the mice were divided into four groups (n = 4): HepG2/sorafenib (Control), Apatinib, Apatinib + SP600125, and Apatinib + PD98059 groups. For the apatinib group, apatinib was administered orally at a dose of 150 mg/kg once a day by gavage. For the apatinib + SP600125 or PD98059 group, after oral administration of apatinib, the mice were injected with SP600125 or PD98059 at 10 mg/kg every 3 days. The mice were sacrificed after about 4 weeks of treatment and tumors were collected. Subsequently, tumor weight and volume were examined. Tumor volume = 0.52 ×  length × width^2^. For the western blot, the tumor tissues were cut into pieces and mixed with RIPA lysis buffer and then homogenized to extract total proteins. The animal research was approved by the Ethics Committee of Shandong First Medical University (No. KY-2021-16).

### Statistical methods

All the experiments were performed in triplicates. Data processing was conducted using GraphPad Prism (Version 8.3.0, GraphPad, San Diego, CA, USA). The results were presented as mean ± SD and subjected to *t*-tests for comparisons between the two groups. A significance level of *p* < 0.05 was considered statistically significant.

## Results

### The microtubule disease and epithelial-mesenchymal transformation (EMT) changes in sorafenib-resistant HCC

**Figure 1:**
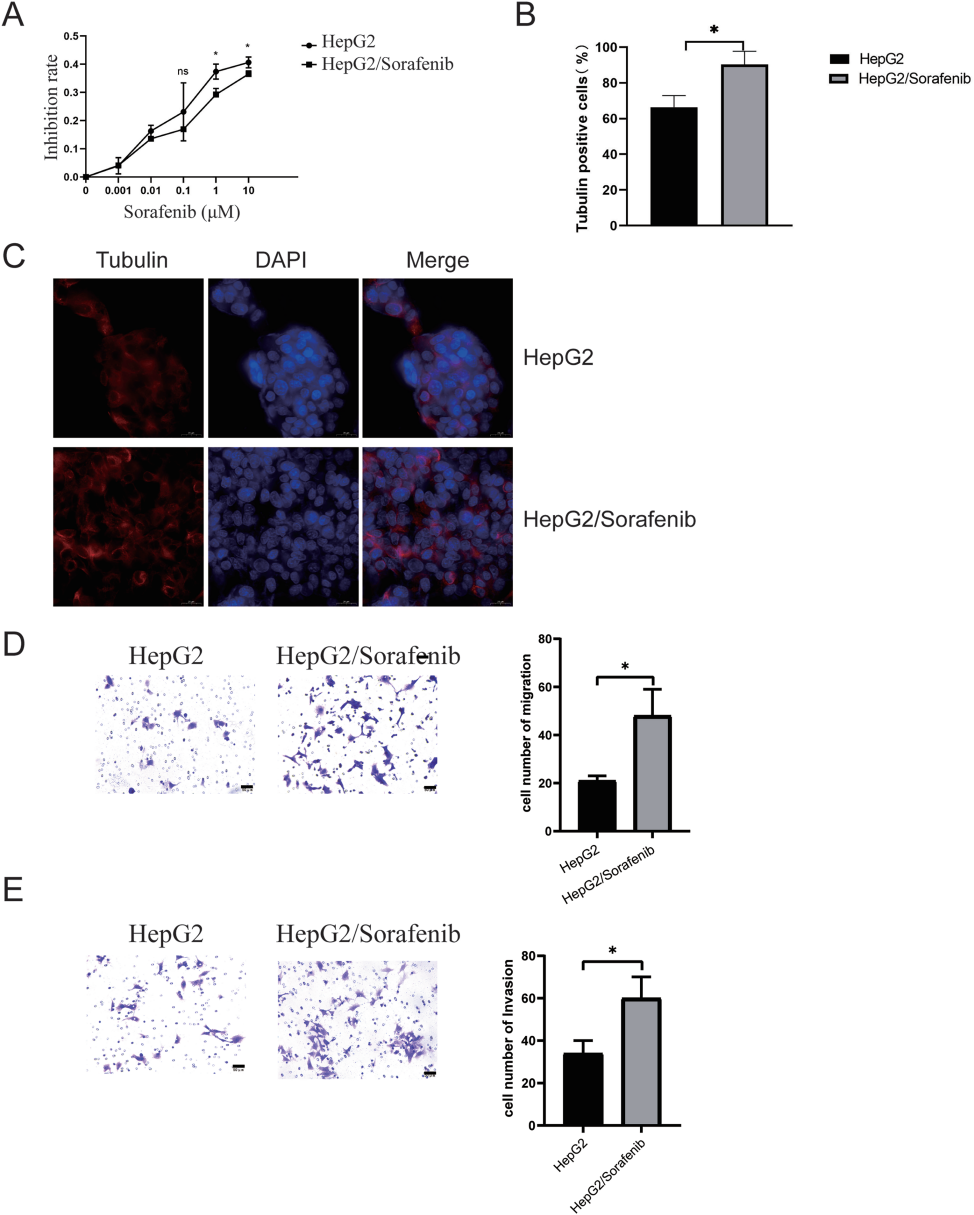
The microtubule disease change in sorafenib-resistant hepatocellular carcinoma cells. (A) CCK-8 assays for drug resistance. (B and C) Immunofluorescence for microtubule structure. Scale bar: 50 μm. (D) Transwell assay for migration. Scale bar: 50 μm. (E) Transwell assay for invasion. Scale bar: 50 μm. Values were shown as mean ± SD. Differences between the two groups were analyzed using a two-tailed *t*-test. *p < 0.05 *vs*. HepG2 group.

Sorafenib was introduced to both HepG2 and HepG2/Sorafenib cells to assess drug resistance in HCC. As illustrated in Fig. 1A, HepG-2/Sorafenib exhibited greater resistance to sorafenib compared to HepG2. Microtubule structures are associated with sorafenib resistance [21]. Thus, microtubule structures were observed in both cell lines. And there was an obvious change in microtubule structures between HepG2 and HepG2/Sorafenib groups (Fig. 1B and C). Notably, drug-resistant strains demonstrated significantly heightened migration and invasive capabilities compared to normal cancer cells (*p* < 0.05, respectively, Fig. 1D and 1E).

**Figure 2:**
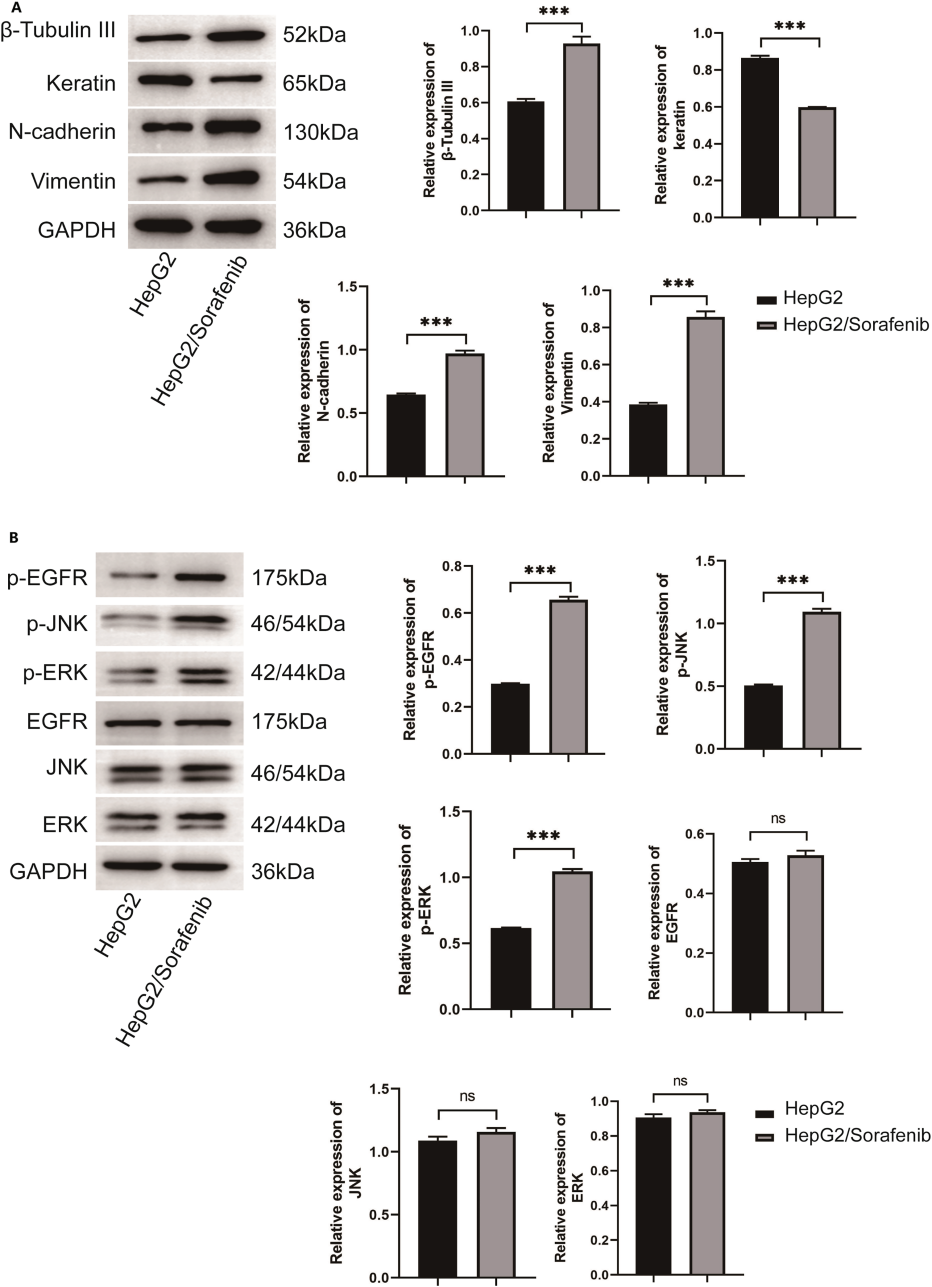
The EMT change in sorafenib-resistant hepatocellular carcinoma cells. (A) Western blot was used to detect the expression of EMT related protein. (B) Western blot was used to detect the expression of EGFR/JNK/ERK signaling pathway related protein. Values were shown as mean ± SD. Differences between two groups were analyzed using a two-tailed *t*-test. ***p < 0.001 *vs*. HepG2 group. ns, not significant.

In consideration of the importance of EMT in sorafenib resistance of HCC, EMT-associated proteins including β-Tubulin III, N-cadherin, and Vimentin were detected. In addition, during the process of EMT in tumor cells, keratin expression would be suppressed and replaced by Vimentin expression [22]. As shown in Fig. 2A, the expression levels of β-Tubulin III, N-cadherin and Vimentin were markedly elevated in HepG2/Sorafenib compared to HepG2, whereas keratin expression was lower in the drug-resistant cells (*p* < 0.05, respectively). As EGFR/JNK/ERK signaling was highly correlated with sorafenib resistance in HCC, the phosphorylation of EGFR, JNK and ERK were also examined. As Fig. 2B displayed, the phosphorylation of the three proteins were obviously promoted in HepG2/sorafenib cells. These findings implied that sorafenib-resistant HCC was associated with EMT changes and EGFR/JNK/ERK signaling.

### Effect of apatinib on the bioactivity of sorafenib resistant HCC

**Figure 3:**
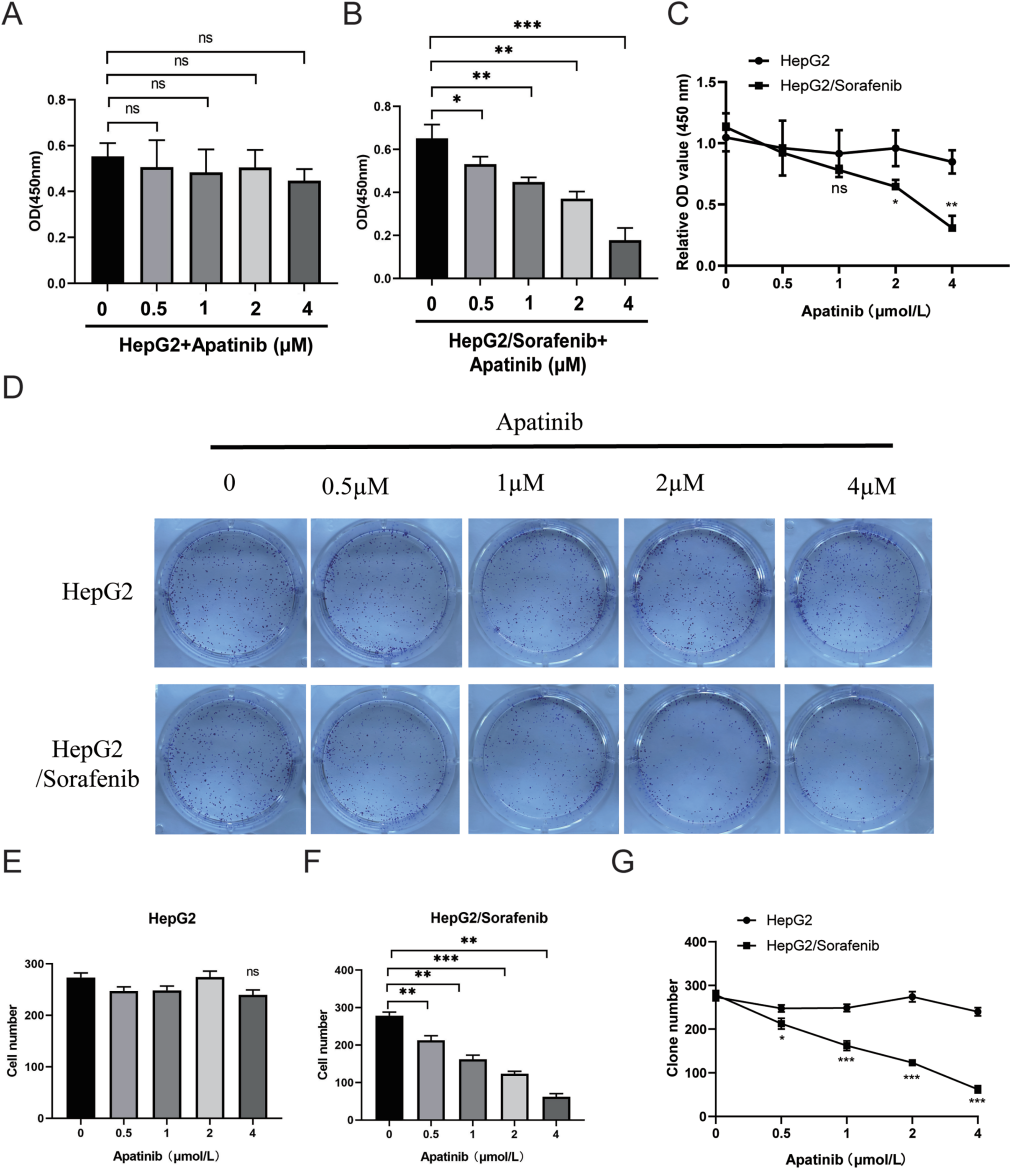
Apatinib inhibited HepG2/Sorafenib cell viability and proliferation in a concentration-dependent manner. (A and B) CCK-8 assay was used to examine the effects of apatinib on proliferation of HepG2 (A) and HepG2/Sorafenib (B) cells; (C) The compasion of proliferation between HepG2 and HepG2/Sorafenib groups; (D) Colony formation assay; (E and F) Quantitative results of colony formation in HepG2 (E) and HepG2/Sorafenib (F) groups; (G) The comparison of colony formation between HepG2 and HepG2/Sorafenib groups. Values were shown as mean ± SD. Differences between two groups were analyzed using a two-tailed *t*-test. *p < 0.05, **p < 0.01, ***p < 0.001 *vs*. HepG2/Sorafenib group. ns, not significant.

**Figure 4:**
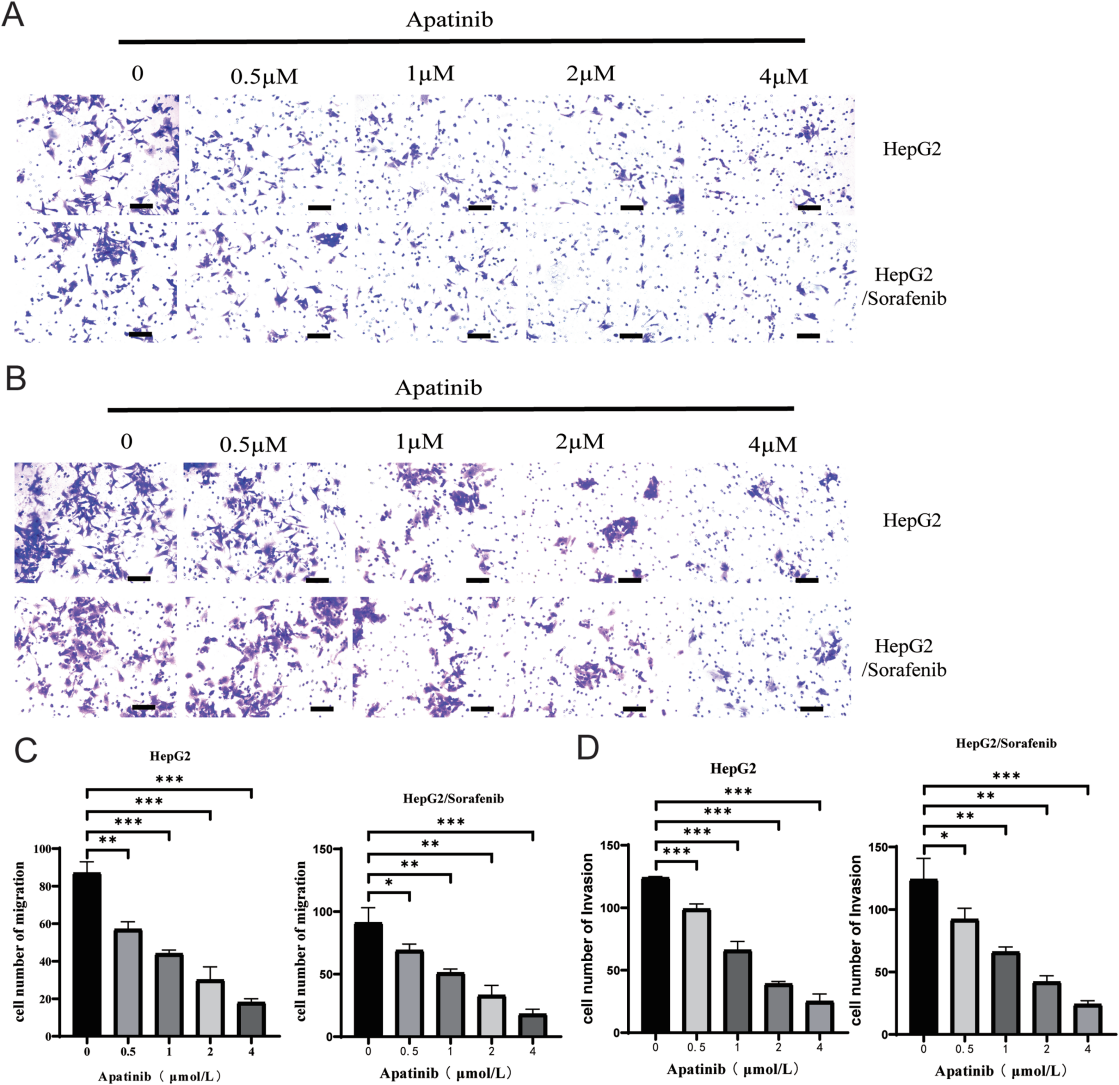
Apatinib inhibited HepG2/Sorafenib migration and invasion in a concentration-dependent manner. (A) Transwell assay for migration. Scale bar: 50 μm; (B) Transwell assay for invasion. Scale bar: 50 μm; (C) Quantitative results of the migration of HepG2 and HepG2/Sorafenib cells; (D) Quantitative results of the invasion of HepG2 and HepG2/Sorafenib cells. Values were shown as mean ± SD. Differences between two groups were analyzed using a two-tailed *t*-test. *p < 0.05, **p < 0.01, ***p < 0.001 *vs*. HepG2 group or HepG2/Sorafenib group.

Next, we examined the effects of apatinib on the malignant phenotypes of sorafenib-resistant HepG2 cells and HepG2 cells. Within HepG2/Sorafenib cells, the groups treated with apatinib exhibited a notable dose-dependent reduction in proliferation, colony formation, migration and invasion capabilities compared to the blank group (Fig. 3A–G and Fig. 4A–D, *p* < 0.05, respectively). However, the impact of apatinib on HepG2 cell viability and proliferation was minimal (Fig. 3A and E), while the migration and invasion of HepG2 cells were obviously inhibited with apatinib treatment (Fig. 4A–D). These observations collectively suggested that apatinib hindered the bioactivity of HepG2/Sorafenib cells in a concentration-dependent manner, and the inhibitory effects were similar to those of HepG2 cells.

### Effect of apatinib on the expression of EMT-related proteins in sorafenib resistant HCC cells

**Figure 5:**
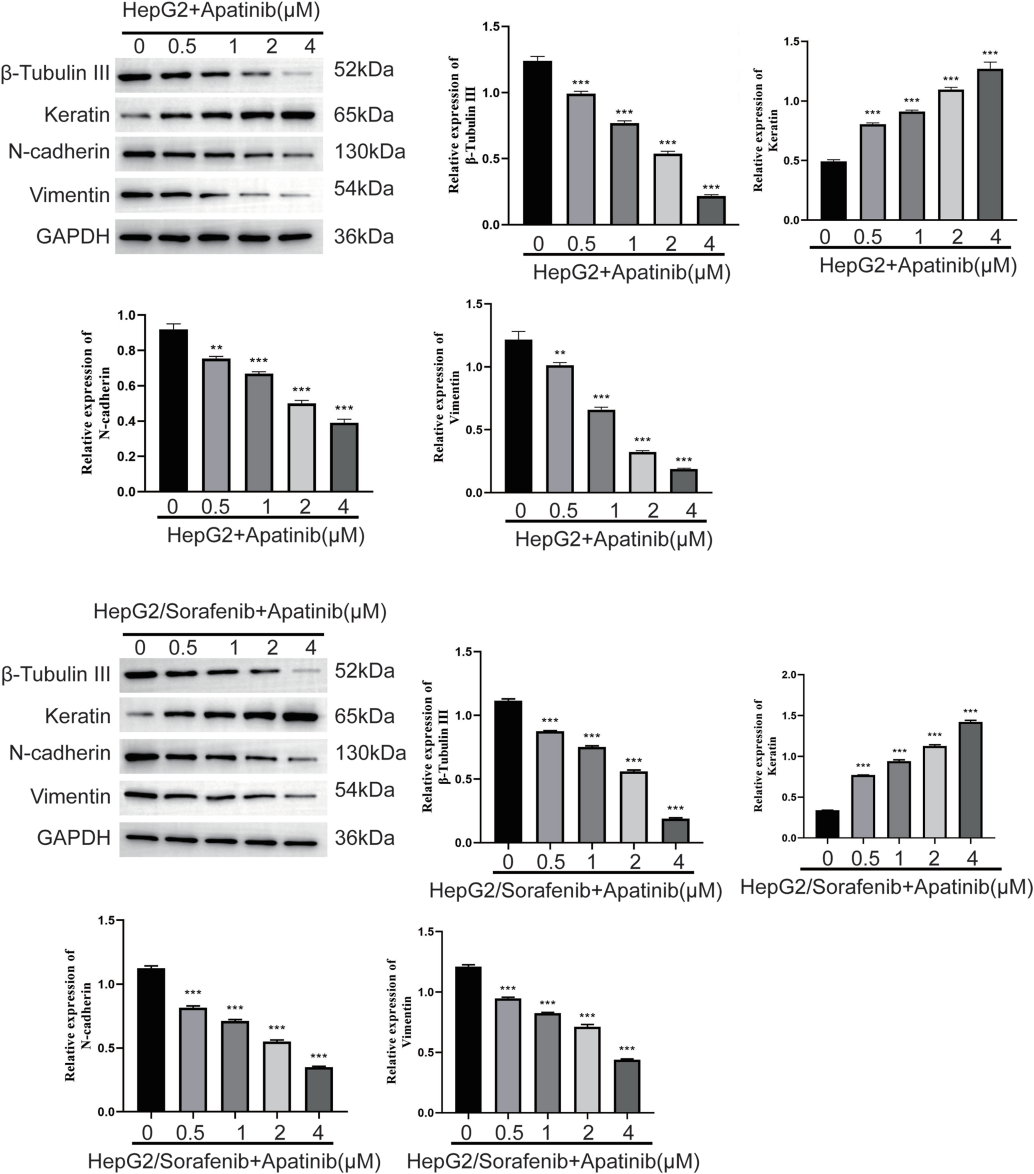
Effect of apatinib on the expression of EMT related proteins in sorafenib-resistant hepatocellular carcinoma cells. Values were shown as mean ± SD. Differences between two groups were analyzed using a two-tailed *t*-test. **p < 0.01, ***p < 0.001 *vs*. HepG2 group or HepG2/Sorafenib group.

Further, to confirm whether EMT was associated with the effects of apatinib on sorafenib-resistant HepG2 cells, we examined the expression of EMT-associated proteins after apatinib treatment. As depicted in Fig. 5, apatinib demonstrated a concentration-dependent inhibition of β-Tubulin III, N-cadherin, and Vimentin expression while promoting keratin expression in HepG2/Sorafenib and HepG2 cells (*p* < 0.05, respectively, Fig. 5). Besides, the impact of apatinib on the four EMT-associated proteins in HepG2/Sorafenib cells was similar to that of HepG2 cells (*p* < 0.05, respectively). These results indicated that apatinib exerted a pronounced effect on the expression of EMT-related proteins in sorafenib-resistant cells similar to that of sorafenib-sensitive HepG2 cells.

### Effects of Apatinib on EGFR/JNK/ERK signaling pathway in sorafenib-resistant HCC cells

**Figure 6:**
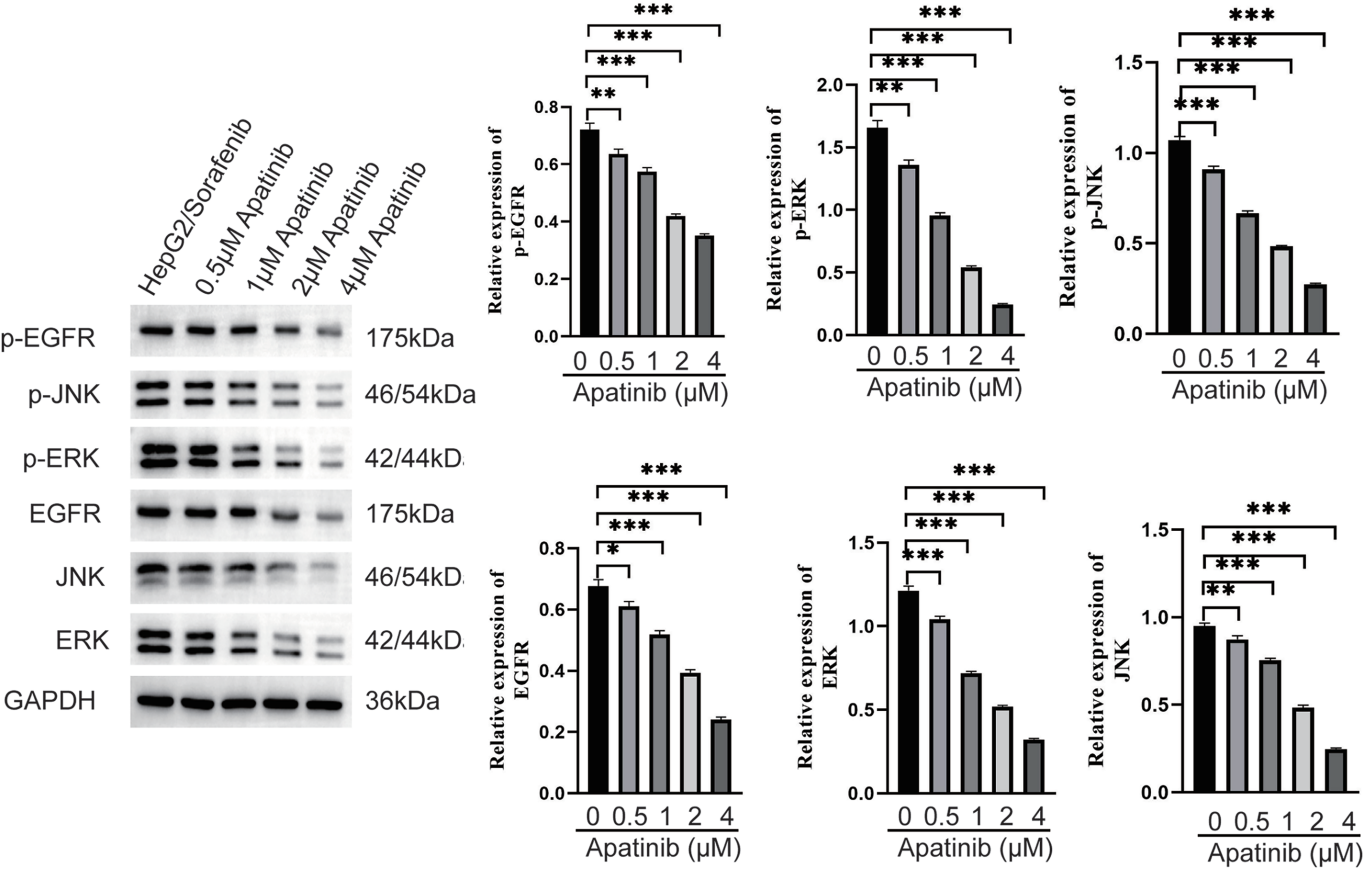
Effect of apatinib on the expression of EGFR/JNK/ERK signaling pathway related proteins in sorafenib-resistant hepatocellular carcinoma cells. Values were shown as mean ± SD. Differences between two groups were analyzed using a two-tailed *t*-test. *p < 0.05, **p < 0.01, ***p < 0.001 *vs*. HepG2/Sorafenib group.

**Figure 7:**
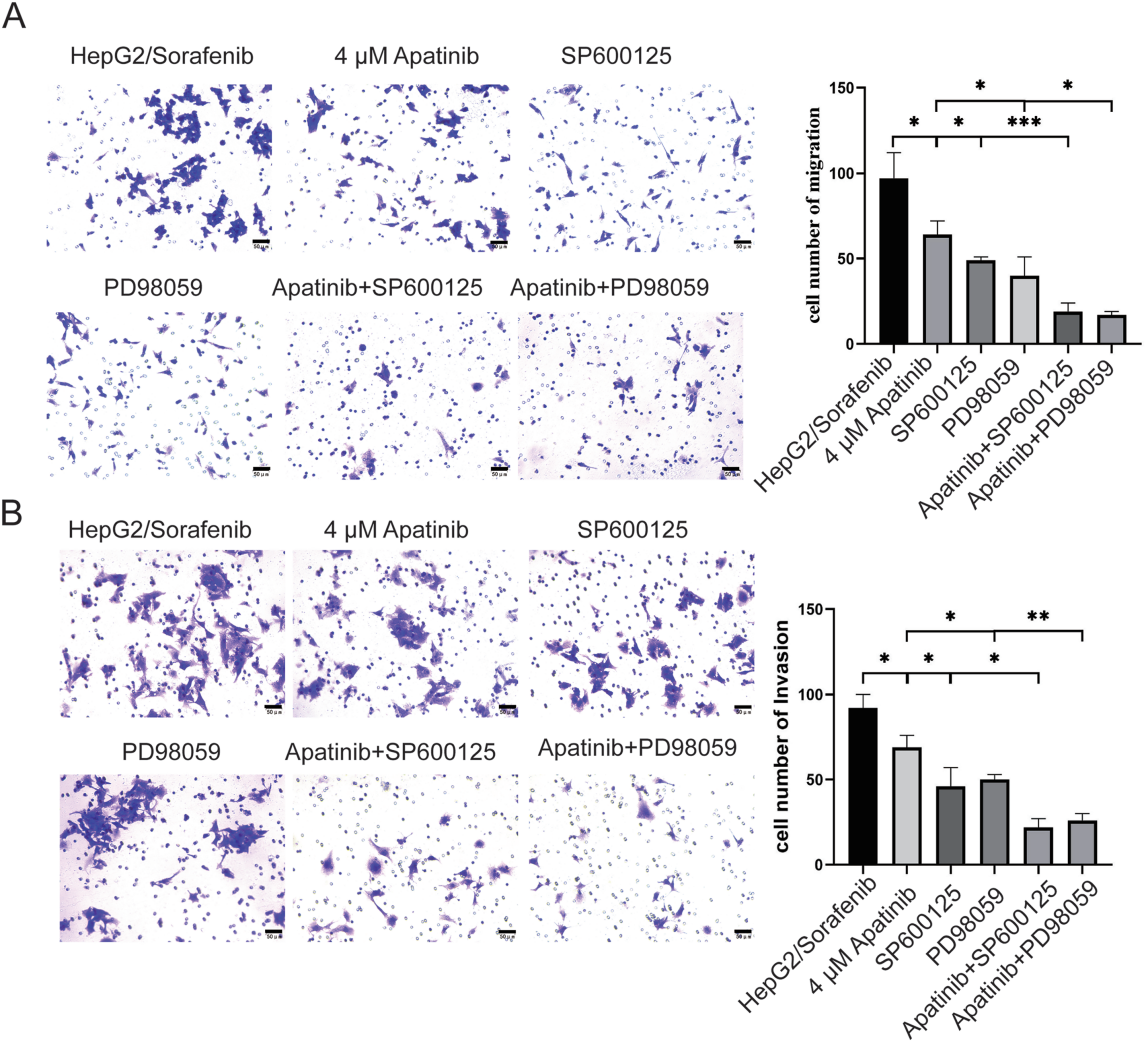
SP600125 and PD98059 promote apatinib inhibition of migration and invasion of HepG2/Sorafenib cells. (A) Transwell assay for migration. (B) Transwell assay for invasion. Values were shown as mean ± SD. Differences between two groups were analyzed using a two-tailed *t*-test. *p < 0.05, **p < 0.01, ***p < 0.001.

To further elucidate the roles of apatinib in the EGFR/JNK/ERK signaling pathway in sorafenib-resistant HCC, HepG2/Sorafenib cells were exposed to varying apatinib doses, followed by the addition of JNK- and ERK-specific inhibitors, SP600125 and PD98059, for mechanistic exploration. Compared to the HepG2/Sorafenib group, the protein expression of p-EGFR, p-JNK, p-ERK, EGFR, JNK, and ERK significantly decreased in the different apatinib dose groups (*p* < 0.05, respectively, Fig. 6). Furthermore, SP600125 and PD98059 enhanced apatinib’s inhibition of migration and invasion in resistant strains (*p* < 0.05, respectively, Fig. 7A and B).

**Figure 8:**
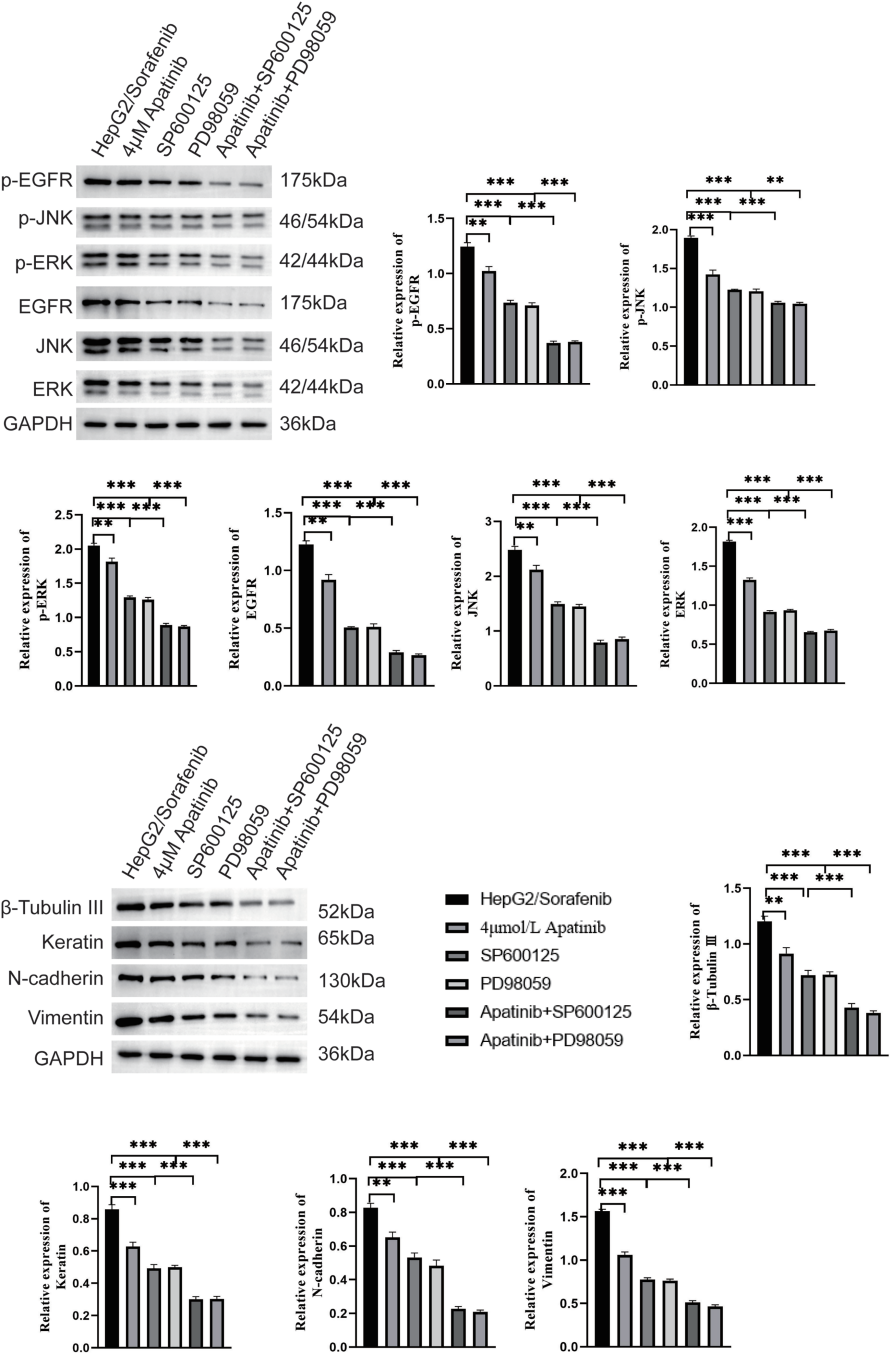
SP600125 and PD98059 contribute to the inhibition of EMT and EGFR/JNK/ERK pathway related protein by apatinib in HepG2/Sorafenib cells. Values were shown as mean ± SD. Differences between two groups were analyzed using a two-tailed *t*-test. **p < 0.01, ***p < 0.001.

Additionally, SP600125 and PD98059 demonstrated a promoting effect on the inhibition of p-EGFR, p-JNK, p-ERK, EGFR, JNK, ERK, β-Tubulin III, N-cadherin, and Vimentin protein expression by apatinib in HepG2/Sorafenib cells (*p* < 0.05, respectively, Fig. 8). In summary, these *in vitro* results indicated that apatinib inhibited the malignancy of sorafenib-resistant HCC by inhibiting EMT and the EGFR/JNK/ERK pathway.

### Apatinib affects development of sorafenib-resistant HCC in vivo

**Figure 9:**
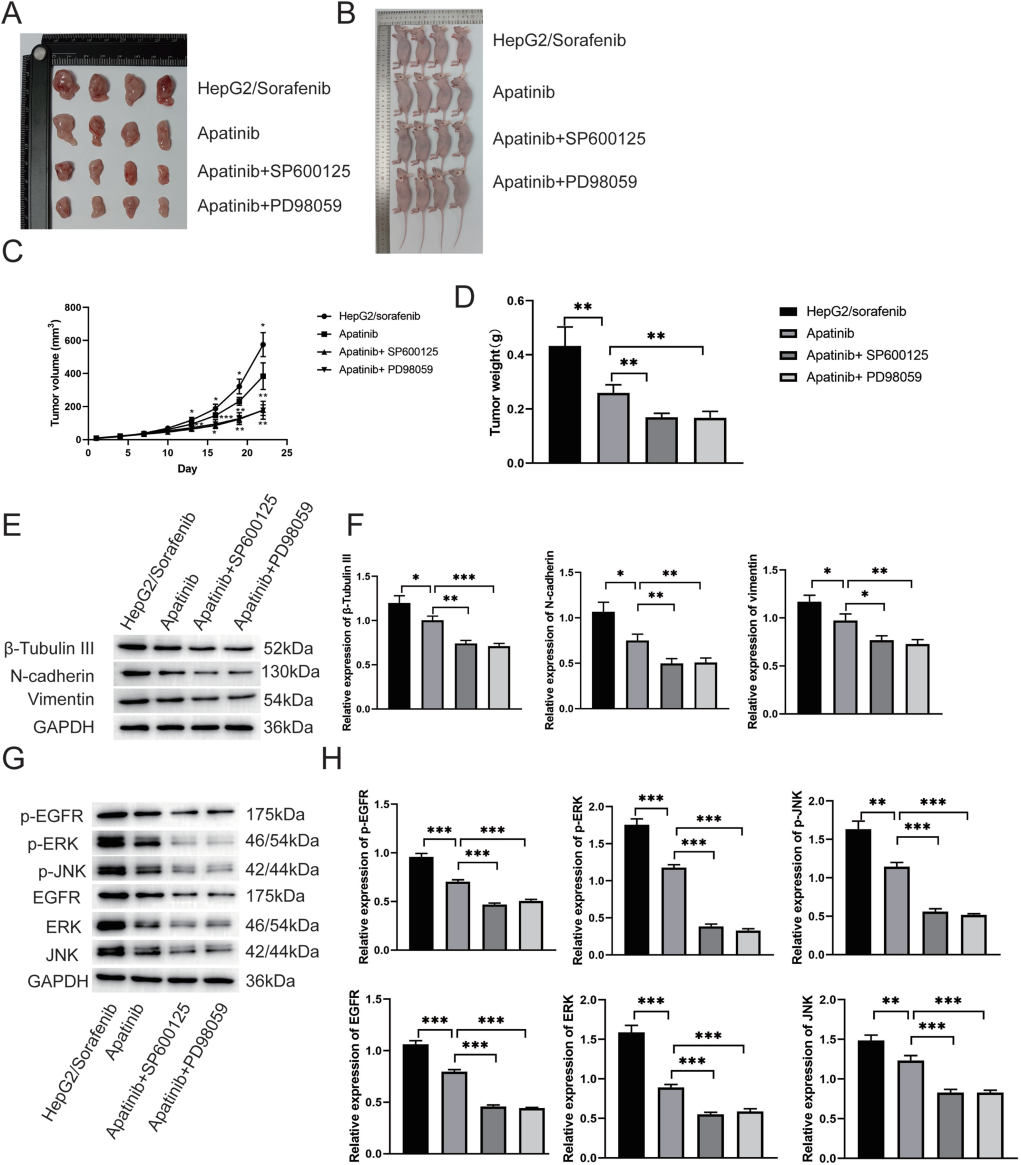
*In vivo* experiments proved that apatinib alleviated sorafenib resistance of HCC by inhibiting EMT and the EGFR/JNK/ERK pathway. (A and B) The images showed the tumors (A) and mice (B) in HepG2/Sorafenib, apatinib, apatinib + SP600125 and apatinib + PD98059 groups. (C and D) The quantitative results of tumor volume and tumor weight; (E and F) Effect of apatinib on the expression of EMT related proteins, including β-tubulin III, N-cadherin and Vimentin; (G and H) Effect of apatinib on the expression of p-EGFR, p-ERK, p-JNK, EGFR, ERK and JNK. Values were shown as mean ± SD. Differences between two groups were analyzed using a two-tailed *t*-test. *p < 0.05, **p < 0.01, ***p < 0.001.

To explore the effects of apatinib on sorafenib-resistant HCC *in vivo*, the HepG2/sorafenib cells were subcutaneously injected into nude mice. As shown in Fig. 9A–D, apatinib treatment inhibited the growth of tumor compared to HepG2/sorafenib group (*p* < 0.01). And SP600125 and PD98059 enhanced the inhibitory effects of apatinib in tumor growth in HCC (*p* < 0.01). Similarly, apatinib treatment inhibited the expression of EMT-associated proteins including β-Tubulin III, N-cadherin and Vimentin *in vivo* (Fig. 9E and F). And SP600125 or PD98059 treatment further reduced the expression of three proteins (Fig. 9E and F). Then similar effects of apatinib on p-EGFR, p-JNK, p-ERK, EGFR, JNK, ERK (Fig. 9G and H). Therefore, the *in vivo* experiments similarly proved that apatinib alleviated the development of sorafenib-resistant HCC by inhibiting EMT and the EGFR/JNK/ERK pathway.

## Discussion

Currently, targeted therapy stands out as a predominant approach in liver cancer [23,24], with sorafenib emerging as a leading multikinase inhibitor in this domain. As a novel multi-targeted anti-tumor drug, sorafenib adeptly hinders tumor angiogenesis and restrains tumor cell replication, effectively impeding tumor growth [13]. Despite notable progress in cancer therapeutics that has enhanced clinical outcomes, drug resistance remains a significant challenge [25,26]. Fortunately, *in vivo* experiments showed that apatinib exhibited antiangiogenic and antitumorigenic effects in HCC and drug safety evaluation indicated that mice treated with sorafenib exhibited diarrhea, skin rash, and reduced body weight, while these adverse effects were not detected in those treated with apatinib [27]. Additionally, apatinib has been reported to improve overall survival of patients with sorafenib-resistant HCC [16,28,29]. Similarly, in our study, apatinib inhibited the growth and movement of HepG2/sorafenib cells. Besides, apatinib exerted an inhibitory effect on the movement of HepG2 cells. Of note, the effects of apatinib on HepG2/sorafenib cells were more obvious than those in HepG2 cells.

In the context of resistance development, EMT serves as a crucial determinant, orchestrating the transformative “switch” of cancer cells from a quiescent to a proliferative state. Approximately 90% of cancer patients eventually suffer from the growth of metastatic tumors beyond the primary site [26,30]. Throughout tumor onset and progression, cancer cells derived from epithelial cells undergo EMT, gaining motility and invasive capabilities and disseminating through the bloodstream or lymphatic system to distant sites [31].

EMT involves a gradual loss of epithelial characteristics in cancer cells originating from epithelial cells, leading them to adopt a more mesenchymal-like state. This transformation enhances cancer cells’ ability to invade surrounding tissues and spread to distant parts of the body [32]. During EMT, increased expression of N-cadherin results in the loss of intercellular adhesion and heightened cell motility, facilitating tumor cell invasion [33]. Notably, β-tubulin III serves as the exclusive marker for resistance to microtubule-targeting drugs [34]. And keratin expression is decreased and replaced by Vimentin expression in EMT [22].

Multiple studies have indicated that sorafenib resistance promoted EMT in HCC cells [35]. Similarly, the present study revealed that the expression levels of β-Tubulin III, N-cadherin, and Vimentin were significantly higher in HepG2/Sorafenib compared to HepG2 cells. Conversely, keratin expression was lower in the drug-resistant cells. The results indicated that EMT process may participate in sorafenib resistance of HCC. After apatinib treatment, the expression levels of EMT-related proteins were obviously reduced in sorafenib-resistant HepG2 cells similar to those in HepG2 cells. Therefore, apatinib exerted inhibitive effects on sorafenib-resistant HCC partly through EMT process.

Given the intricate link between EMT and tumor infiltration and migration, contemporary researchers are committed to unraveling the complexities of cancer dissemination by delving into the underlying molecular mechanisms of EMT. Studies indicate that various signals can trigger the EMT process [36–38]. The EGFR, a glycoprotein in the tyrosine kinase receptor family, resides on the cell membrane surface and becomes activated upon ligand binding. EGFR activates intracellular kinase pathways, such as ERK, by binding with ligands and inducing cell proliferation [39].

Research points to the involvement of the EGFR/JNK/ERK signaling pathway in regulating chemoresistance mechanisms in gastric cancer; however, reports on its role in liver cancer resistance studies are limited [40]. In this study, we observed that the expression levels of p-EGFR, p-JNK, and p-ERK were significantly higher in HepG2/Sorafenib compared to HepG2 cells. This observation suggests that the EGFR/JNK/ERK pathway plays a role in the mechanism of sorafenib resistance in HCC. Apatinib, an anti-angiogenic targeted drug, has demonstrated the ability to reverse drug resistance in cancer cells [41]. Nonetheless, its impact on the EGFR/JNK/ERK signaling pathway in HCC resistance remains unexplored. In light of this, JNK- and ERK-specific inhibitors, SP600125 and PD98059, were employed, and the results suggest that apatinib exerted an inhibitory effect on the progression of sorafenib-resistant HCC by inhibiting both EMT and the EGFR/JNK/ERK pathway.

This study speculated that apatinib could ameliorate sorafenib-resistant HCC by modulating EMT and EGFR/JNK/ERK pathway, which is of great significance. The study provided a new perspective for exploring treatment strategies for sorafenib-resistant HCC. The knowledge gaps include that the proposed mechanism needs further verification through *in vitro*, *in vivo* and clinical studies, and other interacting pathways or factors remain to be explored. To address these, large-scale clinical trials and relevant experiments can be conducted. In the next five years, it’s expected that more in-depth preclinical studies will be carried out to clarify the molecular mechanisms using multi-omics technologies. The number of relevant clinical trials will increase, helping to formulate treatment guidelines. Moreover, new drug targets or biomarkers may be discovered, promoting the development of precision medicine and enhancing the understanding and management of sorafenib-resistant HCC.

## Conclusion

In conclusion, this study revealed that sorafenib-resistant HCC exhibited both microtubule abnormalities and EMT changes. Considering that apatinib influences sorafenib-resistant HCC by inhibiting the EGFR/JNK/ERK signaling pathway, it is plausible to speculate that apatinib could inhibit the development of sorafenib-resistant HCC through the EMT process and EGFR/JNK/ERK pathway. These findings hold significant implications for comprehending the sorafenib resistance process in HCC and provide insights into the mechanism by which apatinib operates in sorafenib-resistant HCC.

## Data Availability

The data that support the findings of this study are available from the corresponding author upon reasonable request.
